# Expression and function of NET-1 in human skin squamous cell carcinoma

**DOI:** 10.1007/s00403-013-1423-9

**Published:** 2013-11-07

**Authors:** Jie zhang, Jianli Wang, Li Chen, Guilan Wang, Jing Qin, Yuyin Xu, Xingyu Li

**Affiliations:** 1 Dermatology Department, Affiliated Hospital of Nantong University, Nantong, 226001 People’s Republic of China; 2 Department of Pathology, School of Medicine, Nantong University, Nantong, 226001 People’s Republic of China

**Keywords:** Skin squamous cell carcinoma (SSCC), NET-1 (TSPAN1, TSPAN1/C4–8), RNA interference (RNAi)

## Abstract

To evaluate the clinicopathological significance of NET-1 in human skin squamous cell carcinoma (SSCC). The expression of NET-1 and Ki67 protein was detected using immunostaining from 60 SSCC cases, 50 SIN samples and ten normal skin tissues. The vectors expressing NET-1, siRNA NET-1 and shRNA NET-1 were constructed, as well as negative controls (target-off). In transfected A431 cells, the expression of NET-1 was detected by qRT-PCR, Western blot and immunofluorescence staining; the proliferation and migration of cells was evaluated by MTT, flow cytometry, wound healing and transwell chamber assays. The stable cell lines transfected with shRNANET-1 was inoculated in nude mice for in vivo study. (1) The levels of NET-1 were significantly higher in SSCC (96.67 %) and SIN III (93.75 %) than that in SIN I and II (41.18 %), (*P* < 0.05). NET-1 expression was significantly enhanced in spindle-cell SSCC (75 %) versus other histological types (*P* < 0.05). (2) The expression of NET-1 in A431 cells transfected with siRNANET-1 or shRNANET-1 was significantly decreased; the proliferation and migration of these cells were obviously inhibited as compared to controls (*P* < 0.05). (3) The growth of subcutaneous tumors was significantly inhibited associated with reduction in the expression of NET-1 vs. the negative control or untreated group (*P* < 0.05). The overexpression of NET-1 in tumor cells may be closely related to the malignant phenotype of SSCC. NET-1 RNAi used in this study can specifically and effectively downregulate NET-1 gene expression; thus SSCC proliferation, invasion and tumor growth were attenuated. NET-1 might be one of the potential targets for SSCC therapy.

## Introduction

Skin squamous cell carcinoma (SSCC) is among the top three common skin cancers, ranking behind basal cell carcinoma (BCC) and ahead of melanoma. SSCC is a significant public health problem despite their low mortality rate [23]. Most cases arise from the sun-exposed skin of elderly people, because the solar radiation causes DNA alterations and mutations in DNA replication. A number of potentially useful molecular markers or tests have been proposed, these include some novel genes and proteins closely related to the carcinogenesis, development and prognosis [11, 16, 22], which may be of great significance for early diagnosis and treatment of SSCC.

NET-1 (or TSPAN1, TSPAN1/C4-8, Gene ID: 10103) and NET2-7 are called NET-X, which are members of seven transmembrane four superfamily (TM4SF) found by Serru in 2000 in EST database [25, 27] TM4SF is a group of proteins containing four hydrophobic transmembrane domains forming two extracellular loop structures with different size. Its N-terminal and C-terminal were located in the cytoplasm. Currently, TM4SF members have increased to 25, although their amino acid sequence share a high homology, there are significant differences between different tissues and cells, particularly the recently reported TM4SF molecules, such as CD9, CD63, CD82, CO-029, PETA-3/SFA-1, SAS, etc., which have been identified as tumor-associated proteins[14, 18, 25, 27]. NET-1 gene is located at chromosome 1 p34.1, and its mRNA is 1,297 bp long encoding a protein with 241 amino acids. In prior study from this laboratory, NET-1, as a new member of TM4SF plays an important role in cell signal transduction [8, 9, 25, 27], and is closely correlated with proliferation, invasion and poor prognosis of cancers [3, 5, 7–10]. NET-1 has been identified through the serial analysis of gene expression (SAGE) database as overexpressed in breast and gastric adenocarcinoma cells [1, 15]. The NET1 gene has a key role in organization of the actin cytoskeleton and thus in the ability of cancer cells to migrate and invade. We found that *NET*-*1* was frequently expressed at a higher level in hepatocellular carcinoma tissue than in peritumoral tissue [6]. Thus, *NET*-*1* may be a suitable target for gene therapy. Thus far, to our knowledge, there have not been any reports investigating the delivery of *NET*-*1* siRNA into human skin squamous cells.

RNA interference (RNAi) has already become an effective way to identify gene function. The mechanism for RNAi function to silence a target gene is to form a silencing complex (RNA-induced silencing complex, RISC) with the target gene, thereafter degrades its mRNA, resulting in reduction of corresponding protein translation [12, 19].

In this study, we examined the expression of NET-1 protein in human SSCC and skin intra neoplasia (SIN) tissues by immunostaining to assess its clinical and pathological significance. Meanwhile, Ki67 was also detected in these SSCC specimens because it is an important cell proliferation marker which was overexpressed in many cancers and closely correlated with malignant biological behavior and prognosis of many cancers [10, 17]. In addition, we designed shRNA and siRNAs against NET-1, and constructed them into vectors prior to transfected into an epidermoid carcinoma cell line (A431 cells) to identify the effects of NET-1 gene on A431 cells and the growth of transfected A431 cell xenograft in nude mice. Though investigating the expression and function of NET-1 gene, this study will provide a potential target to treat skin carcinoma.

## Materials and methods

### Cases and immunohistochemical staining

A total of 60 SSCC cases, 50 cases of SIN (I–III) and ten specimens from the marginal normal skin of breast cancer as normal controls were provided by department of dermatology, Nantong University Affiliated Hospital, China. The study was approved by the local committee of medical ethics, and written prior informed consent and approval were signed by the participants.

All surgically resected samples were routinely fixed in 40 g/L formaldehyde solution and embedded in paraffin. Immunohistochemical staining was performed to detect the NET-1 and Ki67 expressions in the sections using Dako Elivision TM Plus Two-step System (PV-6000 kit, Zymed, Co. USA). In brief, 4 μm sections were dewaxed in xylene and rinsed in alcohol and graded alcohol/water mixtures. The sections were treated in sodium citrate buffer (10 mM sodium-citrate monohydrate, pH 6.0) in a pressure cooker for antigen retrieval. Subsequently, they were treated again with 0.3 % hydrogen peroxide in absolute methanol to inhibit endogenous peroxidase activity. And then the sections were incubated with diluted normal calf serum to prevent from non-specific staining prior to interaction, with rabbit anti-human polyclonal antibodies against NET-1 (antibody was prepared with the cooperation of San Francisco gene biotechnology Co. USA, dilution 1:200) [3, 5, 7, 10], or mouse anti-human monoclonal antibody Ki67 (Lot:41081001, ZYMED CO,USA, dilution 1:100), overnight at 4 °C. Then, slides were washed with 0.05 % Tween 20 in TBS (pH7.4). Detection was achieved with the DAKO envision+/HRP system (Dakocytomation). The color was developed by 15 min incubation with peroxidase-3,3′-diaminobenzidine (DAB) solution (DAB kit IL1-9032). Finally, sections were weakly counter stained with Mayer’s hematoxylin. Negative controls were made by omitting the primary antibodies. The positive controls were the hepatocellular carcinoma that was proved to express NET-1 and Ki67. Simultaneously, epithelial membrane antigen (EMP) (Lot:41081001, ZYMED CO, USA, dilution 1:100) and higher molecular weight cytokeratin (CK) (Lot:41081001, ZYMED CO,USA, dilution 1:100) were detected to determine tumor original.

All sections were blindly analyzed by two experienced pathologists. Based on the estimated percentages of positive parenchyma cells and/or the immunostaining intensity, which was determined by comparing the immunoreactivity of the positive controls that were included in each experiment, staining results were divided into four categories: (−) positive parenchyma cells were less than 5 % and/or with weakly stained, (+) positive parenchyma cells were ranged from 5 to <50 % and/or with weakly stained, (++) positive parenchyma cells were ranged from 50 to <75 % and/or with moderately stained, and (+++) positive parenchyma cells were more than 75 % and/or with strongly stained [3, 5, 7, 10].

### Plasmid construction

pSilencer 4.1-CMV neo-GFP vector with a neo resistance gene, 4.944 kp and pU6H1-GFP vector (the vector length in the U6 and H1 is 5.1 kp, and there is no extra cloning sequence site in the siRNA fragment between the promoter starting sequence AAAAA and ending sequence TTTTT) were purchased from Ambion Inc. and Qiagen Inc., respectively. According to the NET-1 sequences in the gene bank (library number AF065388), Qiagen siRNA designing software and network computing tools (http://jura.wi.mit. Edu/bioc/siRNA) [2, 13, 20, 21, 26] were used to design siRNA sequence (23 bp) targeting NET-1 and shRNA sequence (55 bp) at 50–100 nt downstream of the mRNA starting codon AUG. The sequence alignment (BLAST) was carried out to ensure specificity and to determine no homology with other human genome. Then, the corresponding DNA sequence was further synthesized by enzyme digestion, ligation, transformation, thus, specific plasmids against siRNA NET-1 and shRNANET-1 were constructed. The random sequences (off-target) in shRNA(shRNA targeting off-T)and siRNA (siRNA targeting off-T) were used as the non-specific negative controls. The sequence and length in recombinant plasmids were shown in Table 1.

**Table 1:** Sequences of shRNA and siRNA expressing insertions

Plasmid Primer	Sequence	Length (bp)
shRNANET-1-S	5′-GATCCCCACAATGGCTGAGCACTTTTCAAGAGAAAGTGCTCAGCCATTGTGGTGA-3′	55
shRNANET-1-AS	3′-GGGTGTTACCGACTCGTGAAAAGTTCTCTTTCACGAGTCGGTAACACCACTTCGA-5′	
siRNANET-1-S	5′-TGTGGTCTTTGCTCTTGGTTTCC-3′	23
siRNANET-1-AS	3′-ACACCAGAAACGAGAACCAAAGG-5	
shRNA off-T-S	5′-GATCCGGAGTACCCTGATGAGATCTTCAAGAGAGATCTCATCAGGGTACTCCTGA-3′	55
shRNA off-T-AS	3′-GCCTCATGGGACTACTCTAGAAGTTCTCTCTAGAGTAGTCCCATGAGGACTTCGA-5′	
siRNA off-T-S	5′-GAGTGATTGGAGGTTGGGGAC-3′	21
siRNA off-T-AS	3′-CTCACTAACCTCCAACCCCTG-5′	

### Cell culture and transfection

A431 cell line (from an epidermoid carcinoma of a 85 old female patient) was a gift from Dr. Gao Tianwen in the military Institute of Dermatology of West Beijing Hospital, Fourth Military Medical University in Xi’an, China. The A431 cells were maintained in DMEM plus 10 % fetal bovine serum and 1 % penicillin/streptomycin (Life Technologies) in a 37 °C, 5 % CO_2_ environment. The cells were transfected with the above plasmids using Lipofectamine™^2000^ transfection reagent (USA Invitrogen Corporation), according to the manufacturer’s instructions. The cells were analyzed at 48 or 72 h after transfection. Cells were used for the below experiments after they were passaged 4–10 times.

### Real-time quantitative polymerase chain reaction (qRT-PCR)

One-hundred twenty-five μg of cDNA per reaction was used for qRT-PCR using SYBR Green reagents and analyzed on ABI Prism 7700 Sequence Detector (Applied Biosystems, Foster City, CA, USA). Upstream and downstream primers of NET-1 0.5 μl (10 pmol/μl) were designed (NET-1-S: 5′-GTGGCTTCACCAACTATACG-3′, NET-1-AS: 5′-GACTGCATTAGTTCGGATGT-3′).All reactions were performed in a 25 μl volume with 150 nM primers, 0.25 units of Amp Erase Uracil-*N*-glycosylase and 1× SYBR Green PCR Master Mix (Applied Biosystems). The thermal cycling profile consisted of a 95 °C denaturation step for 10 min, then 40 cycles at 95 °C for 20 s, 62 °C for 30 s and 72 °C, 30 s annealing extension. All samples were run in duplicate. GAPDH (glyceraldehyde-3-phosphate dehydrogenase) was used as reference gene. (GAPDH-S: 5′-GAAGGTGAAGGTCGGAGTC-3′, GAPDH-AS: 5′-GAAGATGGTGATGGGATTTC-3′). Each was 1.0 μl (6 pmol/μl) and compared to a standard curve created using Universal Human Reference RNA (Stratagene, Cedar Creek, TX, USA). To exclude non-specific amplification and primer–dimer formation, a dissociation curve analysis was performed.

### Western blot

Harvested cells were washed once with PBS, and lysed in Phosphosafe (Novagen, Madison, WI). Protein concentrations were determined by Bradford analysis using Coomassie Plus Protein Assay Reagent (Pierce, Rockford, IL) and equivalent concentration of each sample was mixed with 2× SDS sample buffer [0.1 mol/L Tris–HCl (pH 6.8), 4 % SDS, 20 % glycerol, 0.1 % bromophenol blue, 5 % β- mercaptoethanol], boiling for 5 min, and 10 μl was loaded for electrophoresis. After running in sodium dodecyl sulfate–polyacrylamide gels (5 % spacer gel, 12 % separating gel), semi-dry transfer apparatus (BIO-RAD, Inc.) was used to transfer proteins on the gels onto PVDF membranes. After been blocked with 5 % skim milk, the membranes were incubated with rabbit anti-human NET-1 polyclonal antibody (1:800) overnight at 4 °C.The horseradish peroxidase labeled goat anti-rabbit IgG antibody (1:1,000) was used to probe primary antibody and interact with ECL (Amersham Bioscience). The housekeeping gene product, β-actin, was used to normalize sample proteins.

### Immunofluorescence staining

The transfected cells sticked on the slides were fixed with 4 % paraformaldehyde for 2 h, and then 10 % normal goat serum was added at 37 °C for 1 h, followed by the incubation of rabbit anti-human NET-1 polyclonal antibody (1:200) and mouse anti-human monoclonal antibody ki67 (1:200) at 4 °C overnight. PBS was used as a negative control. After PBS wash three times, FITC-goat-anti-rabbit IgG (1:100) and TRITC-goat-anti-mouse IgG (1:100) were, respectively, incubated in dark at 4 °C overnight. Hochest 33258 was used to stain nuclei for 10 min (final concentration of 50 g/ml), mounted by buffer glycerol and photographed under fluorescent microscope. For FITC, the excitation wavelength is 488 nm; for TRITC, the excitation wavelength is 550 nm; for Hochest, the excitation wavelength is 353.6 nm. The mean ratio was obtained by counting positive cells in at least five random fields per field (magnification 40 × 10).

### MTT assay

At 24 h after transfection, A431 cells in logarithmic growth phase were treated by 0.25 % trypsin and collected, DMEM medium with 10 % newborn calf serum was used to make single cell suspension, and seeded in a 96-well plate by 1 × 10^4^ cells/well, then 150 μl medium was added to each well. The cells were cultured in an incubator at 37 °C containing 5 % CO_2_. The detection was taken at 0, 24, 48 and 72 h. Each well was added with 20 μl MTT (5 g/L, Biobasic Company) solution, cultured for another 4 h, then the medium was discarded, 150 μl DMSO was added to each well for 10 min at 37 °C, gently vibrating to dissolve crystal. The absorbance of each well was measured by microplate reader (wavelength 570 nm).

### Flow cytometry (FCM)

At 48 h after transfection, about 10^6^ cells suspensions were collected and centrifuged at 1,000 rpm for 5 min. The pellets were washed twice with ice-cold 0.01 mol/L PBS and fixed in pre-cooling 70 % ethanol at 4 °C overnight. After washing twice with PBS, cells were incubated in PBS with 5 mg/mL propidium iodide (PI) staining of nuclei and 50 mg/mL RNase A in dark for 30 min at 4 °C. The red fluorescence of DNA-bound PI in cells was measured at 488 nmol/L with a FACS Calibur flow cytometer (Becton–Dickinson, USA). The results were analyzed using the CellQuest software. The percentages of cells in each cycle were measured for each sample. The proliferation index (PI) of each group was calculated, PI = (*S* + G2/*M*)/(G0/G1 + *S* + G2/*M*).

### Cell migration assay by “wound” lane

The cells were seeded into 96-well plates (1.5 × 10^5^/well) the day before transfection allowing their growth up to 100 % confluence (about 6 h after transfection). A straight lane as made on the cell layer using pipet tip to form a “wound” lane. After 24, 48 and 72 h, the relative distances of cell migration from the edges were measured by image software (Image-ProPlus, version 4.5.1, USA) [4].

### Cell migration assay by transwell chambers

Cells were harvested 24 h after transfection; cells were then seeded into 24 transwell inserts (the upper chamber) filled with DMEM without serum. The lower chamber was supplemented with 500 μl DMEM medium with 10 % FCS serum. The upper and lower chambers were separated by an 8 Am pore polycarbonate membrane (Costar, Corning, NY) which was coated with 50 μl of 0.5 mg/ml Matrigel. The cells on the upper surface of the inserts were carefully removed at 36 h after transfection with a cotton swab. Migration cells on the lower surface of the inserts were counted in five random high-powered fields (HPF, 40 × 10) per membrane, and the average number of migrating cells per HPF was calculated.

### Nude mice SSCC xenograft models

To guarantee the cells that were used to develop xenografts in nude mice were stably transfected with shRNA, colony formation assays were performed to select single-cell colonies at 2–3 weeks after growth in soft agar. And then the selected single-cell clones were expanded in the medium containing G418. A431 cells with off-T shRNA control were also selected by G418. The subcutaneous xenografts in nude mice were developed by inoculation of the above cells into animals. Six to eight-week-old specific pathogen-free athymic nude mice (BALB/c Nude, female) were fed with sterilized food, water, and housed in a barrier facility with 12 h light and dark cycles. All procedures were conducted at the animal experiment center of Nantong University, according to guidelines laid out by the Institutional Animal Care and Use Committee. The treated or untreated cells (2 × 10^6^) in 0.2 ml PBS were injected subcutaneously into both sides of the mice nape (*n* = 6 for each group, total 18). The tumor masses were measured every 3 days with a caliper, and tumor volume was calculated by the formula: volume = (1/6) *πab* ^2^ (where a and b represent two perpendicular tumor diameters) [21]. Regression in subcutaneous tumor growth was followed up for 3 weeks. After mice were euthanized, subcutaneous tumors were removed and fixed in buffered formaldehyde (4 % wt/vol in PBS). 4-μm paraffin sections were stained in hematoxylin-eosin (H&E) using standard histological techniques.

Quantification of necrotic area: In sections, the mean size of tumor necrotic area was determined in five random fields under microscope.

### Statistical analysis

Paired data were analyzed using Student’s t test; rank correlation data were analyzed using the Spearman test; relationships between NET-1 expression and Ki67 expression, clinicopathological parameters were analyzed using the *χ* ^2^ test. For all statistical analyses, a difference with *P* < 0.05 was considered significant. In vitro, all tests were transfected into three parallel wells each repeated three times. The untransfected group was taken as a control for comparison. The relative distance of cell migration and migrating cells per transwell chamber membrane, the volume and weight of nude mice xenografts etc., were presented as mean ± SD, respectively. SPSS for Windows (version 13.0, SPSS Inc., Chicago, IL, USA) was used for all statistical analyses.

## Results

### Clinical cases

Of the 60 SSCC cases, 66.67 % of lesions (40 cases) occurred on forehead, face, ears, scalp neck and dorsum of the hands and vermilion part probably due to sunlight exposure; 33.33 % (20 cases) occurred on the soles of the feet and back. Among them, 43 cases were males and 17 females with a median age of 66.7 (range 34–92). None of the patients received chemotherapy or radiotherapy before diagnosis. The average size of the tumor was 2.4 cm (range 1.2–4.6 cm). The clinicopathological features were determined according to WHO histological classification of keratinocytic skin tumors and TNM stage of skin carcinomas [6, 15]. SSCC cases were divided into general SSCC (31 cases, 51.67 %), acantholytic SSCC (10 cases, 16.67 %), spindle-cell SSCC (8 cases, 13.33 %), verrucous SSCC (9 cases, 15 %), and other types (2 cases, 3.33 %) which included pseudovascular SSCC (1 case) and adenosquamous carcinoma (1 case). SIN were divided into I–III, SIN I–II (total 34 cases) attributed to low grade with mild or moderate atypical hyperplasia; SIN III (16 cases) belonged to high grade with atypical keratinocytes involving over 2/3 full-thickness of epidermis and carcinoma in situ. Infiltrating tumor status (T): the size of T1 tumor was 2 cm or less in maximum dimension (stage I 21 cases, 35 %); T2 tumor size was 2–5 cm (27 cases, 45.0 %), and T3 tumor size was over 5 cm in greatest dimension (8 cases, 13.33 %), both T2 and T3 is attributed to the stage II (35 cases, 58.33 %), T4 tumor infiltrated deep extradermal area (1 case, 1.67 %). Tumor with either T4 or regional lymph node metastasis (3 cases, 5.0 %) belonged to stage III (4 cases, 6.67 %). None of the cases was stage IV (tumor with distant metastasis). Grossly, 28.33 % (17 cases) were shallow ulcer, 53.33 % (32 cases) were plaques or nodules, and 18.33 % (11 cases) were cauliflower-like appearance. According to the states s of keratocyte differentiation and dysplasia appearances, the SSCC were divided into well (19 cases, 31.67 %), moderately (29 cases, 48.33 %) and poorly differentiation (12 cases, 20 %). The thickness of tumor was evaluated according to the depth of tumor growth and invasion: <2 mm 28.33 % (17 cases), 2–5 mm 60 % (36 cases), >5 mm 11.67 % (7 cases).

Immunohistochemical results showed: NET-1 and CK were positively located in cytoplasm, EMP positively located in cytomembrane and Ki67 was positively located in the nucleus. All cases from human samples were confirmed from keratinocyte origin by EMA and CK immunostaining. All positive controls expressed NET-1 and Ki67. In contrast, no positive staining was found in negative groups.

### NET-1 expression in SIN and SSCC

**Fig. 1:**
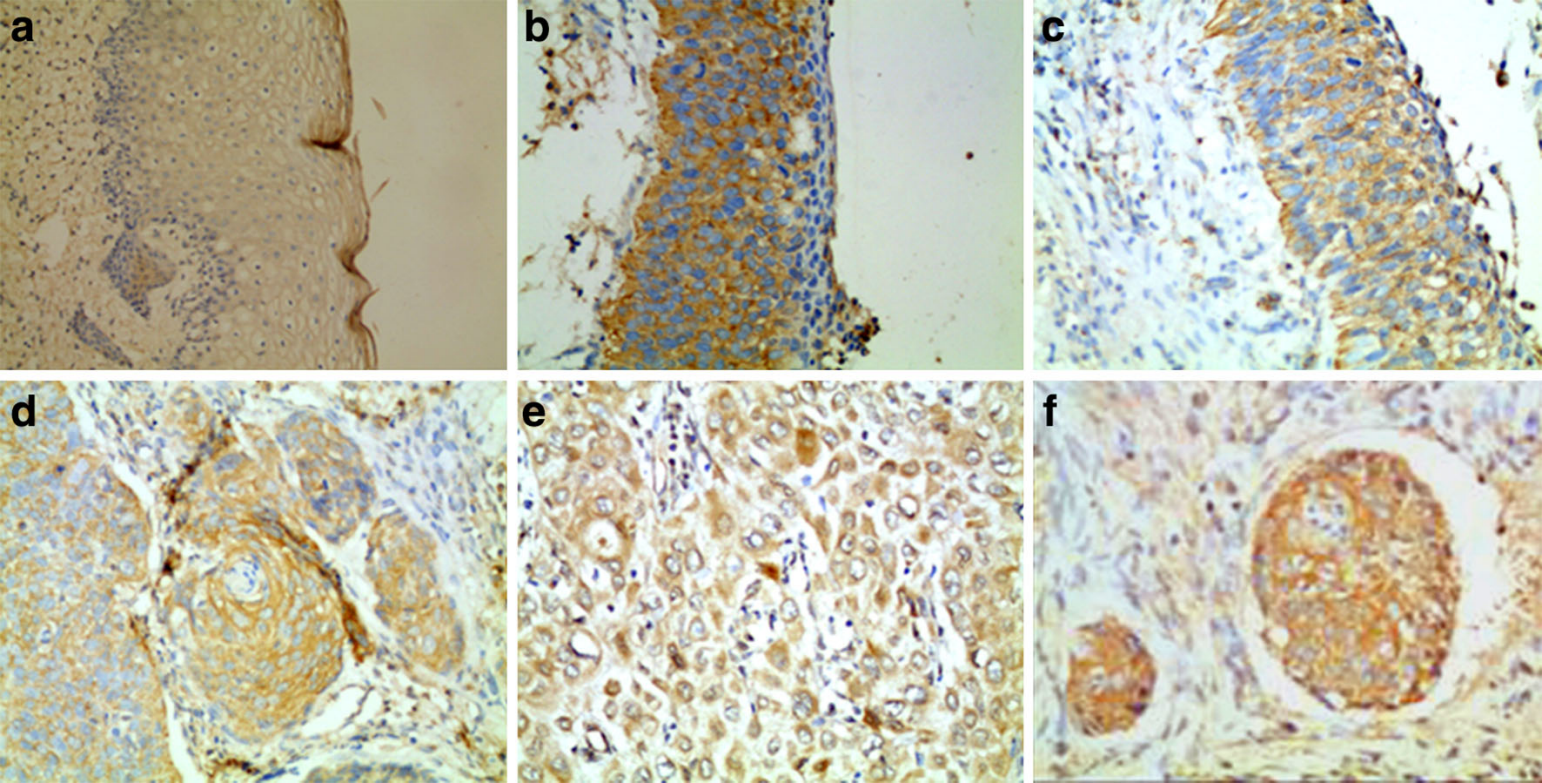
The expressions of NET-1 in SIN and SSCC. Paraffin section of SSCC was stained with anti-NET-1 polyclonal antibody immunocytochemistry methods. NET-1 located in the cytoplasm, *yellow* granulation. NET-1 expressed negatively in normal skin (**a**), evenly in the basal layer and spinous layer in SIN II (**b**), in the full epithelial layer in SIN III(**c**), strongly in SSCC (**d**), especially in lower differentiated (**e**) and metastatic carcinoma emboli of blood vessel (**f**). Magnification ×200

The basal layer in normal skin did not or weakly express NET-1. In contrast, NET-1 was expressed in some SIN cases. In SIN I–II cases, the cells expressing NET-1distributed evenly in the basal layer and spinous layer with weak staining, and in the 2/3 upper epidermis with normal squamous differentiation. The positive rates for NET-1 in SIN I–II were 44.22 % (15/34). In SIN III, the cells with NET-1 were located in the full epithelial layer, and the positive rate was 93.75 % (15/16). In 96.67 % (58/60) SSCC cases, NET-1 was stained intensively with polar disorder, and the expression was enhanced in cells infiltrating stroma and in metastatic emboli in blood vessels (Fig. 1). Comparison of NET-1 expressions between SSCC and SIN was shown in Table 2.

**Table 2:** Comparison of NET-1 expressions between SSCC and SIN I–III

	Cases	NET-1			
		− (%)	+ (%)	++ (%)	+++ (%)
SINI-II	34	20 (58.82)	10 (29.41)	4 (11.76)	0 (0.00)
SINIII	16	1 (6.25)	2 (12.50)	6 (37.5)	7 (43.75)
SSCC	60	2 (3.57)	8 (13.33)	21 (35.0)	29 (48.33)

Chi-square test (Table 2) showed that there was a significant difference in intensity of NET-1 staining among SSCC, SIN III and SIN I–II (*χ* ^2^ = 58.22, *P* < 0.01). NET-1 expression was gradually enhanced from SIN I–II to SIN III and SSCC. Spearman grade analysis suggested an obvious positive correlation among them (*r* = 0.6077, *P* = 0.0000). In intensity of NET-1 staining, there was a significant difference between SIN I–II and SIN III (*χ* ^2^ = 26.9345, *P* < 0.01), but no significant difference between SSCC and SIN III (*P* > 0.05).

### Correlation between NET-1 protein expression and the clinicopathological parameters of SSCC

**Fig. 2:**
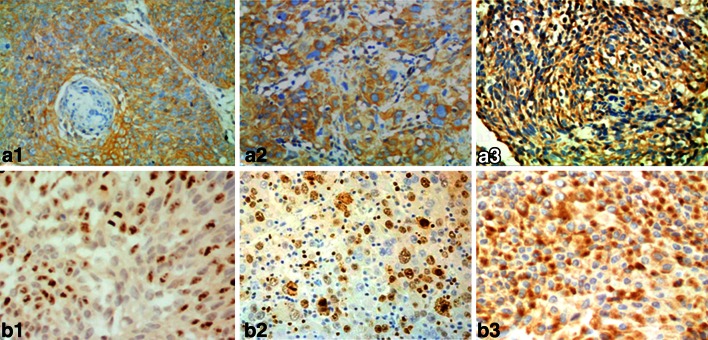
The expressions of NET-1 and Ki67 in SSCC. Paraffin section of SSCC was stained with anti-NET-1 polyclonal antibody (**a**) and Ki67 monoclonal antibody (**b**) immunocytochemistry methods. NET-1 located in the cytoplasm, Ki67 located in the nuclear; **a1**, **b1** well differentiated; **a2**, **b2** middle differentiated and **a3**, **b3** lower differentiated; magnification ×200

The correlation of NET-1 expression with the clinicopathological parameters in SSCC was analyzed (Table 3). There was a correlation in NET-1 expression between Ki67 expression (*χ* ^2^ = 15.2906, *r* = 0.3430, every *P* < 0.05) (Fig. 2), tumor thickness (*χ* ^2^ = 12.8197, *r* = 0.3220, every *P* < 0.05), or cancer differentiation (*χ* ^2^ = 14.3131, *r* = 0.0.3542, every *P* < 0.05). The expression of NET-1 in spindle-cell carcinoma appeared to be more obvious than that in other types, but there was no significant difference between NET-1 expression and SSCC TNM stages, lesion sites, sunlight exposure, gender and age (*P* > 0.05).

**Table 3:** Significant correlation between NET-1 expression and clinical pathological factors in SSCC

	Cases	NET-1			
		− (%)	+ (%)	++ (%)	+++ (%)
Ki67 expression*					
+	12	1 (8.33)	5 (41.67)	3 (25.00)	3 (25.00)
++	14	1 (7.14)	2 (14.29)	5 (35.71)	6 (42.86)
+++	34	0 (0.00)	1 (2.94)	13 (38.24)	20 (58.82)
Thickness*					
<2 mm	17	1 (5.88)	5 (29.41)	8 (47.06)	3 (17.65)
2–5 mm	36	1 (2.78)	3 (8.33)	12 (33.33)	20 (55.56)
>5 mm	7	0 (0.00)	0 (0.00)	1 (14.29)	6 (85.71)
TNM stages					
I	21	1 (4.76)	4 (19.05)	10 (47.62)	6 (28.57)
II	35	1 (5.25)	4 (11.42)	10 (28.57)	20 (57.14)
III	4	0 (0.00)	0 (0.00)	1(0.25)	3 (0.75)
Pathologic type*					
General	31	1 (3.22)	3 (9.68)	10 (32.25)	17 (54.84)
Spinous-release	10	0 (0.00)	2 (20.0)	5 (50.0)	3 (30.00)
Spindle-cell	8	0 (0.00)	0 (0.00)	2 (25.00)	6 (75.00)
Verrucous	9	1 (11.11)	4 (44.44)	2 (22.22)	2 (22.22)
Others	2	0 (0.00)	0 (0.00)	1 (50.0)	1 (50.0)
Gross appearance					
Shallow ulcer	17	1 (5.88)	3 (17.65)	6 (35.29)	7 (41.12)
Plaques or nodules	32	0 (0.00)	4 (12.5)	12 (37.5)	16 (50.00)
Cauliflower like	11	1 (9.09)	1 (9.09)	3 (27.27)	6 (54.55)
Differentiation					
Well	19	2 (10.53)	5 (26.32)	8 (42.11)	4 (21.05)
Moderately	29	0 (0.00)	2 (7.00)	11 (37.93)	16 (55.17)
Poorly	12	0 (0.00)	1 (8.33)	2 (16.67)	9 (75.00)
Sites					
Sunlight exposure	40	2 (5.00)	7 (17.5)	14 (35.00)	17 (42.5)
Non exposure	20	0 (0.00)	1 (5.00)	7 (35.00)	12 (60.0)
Sex					
Male	47	2 (4.25)	5 (10.64)	17 (36.17)	23 (48.94)
Female	13	0 (0.00)	3 (23.08)	4 (30.76)	6 (46.15)

* The difference was significant in groups compared (*P* < 0.05)

### Inhibitory effect of RNAi on NET-1 expression in A431 cells

**Fig. 3:**
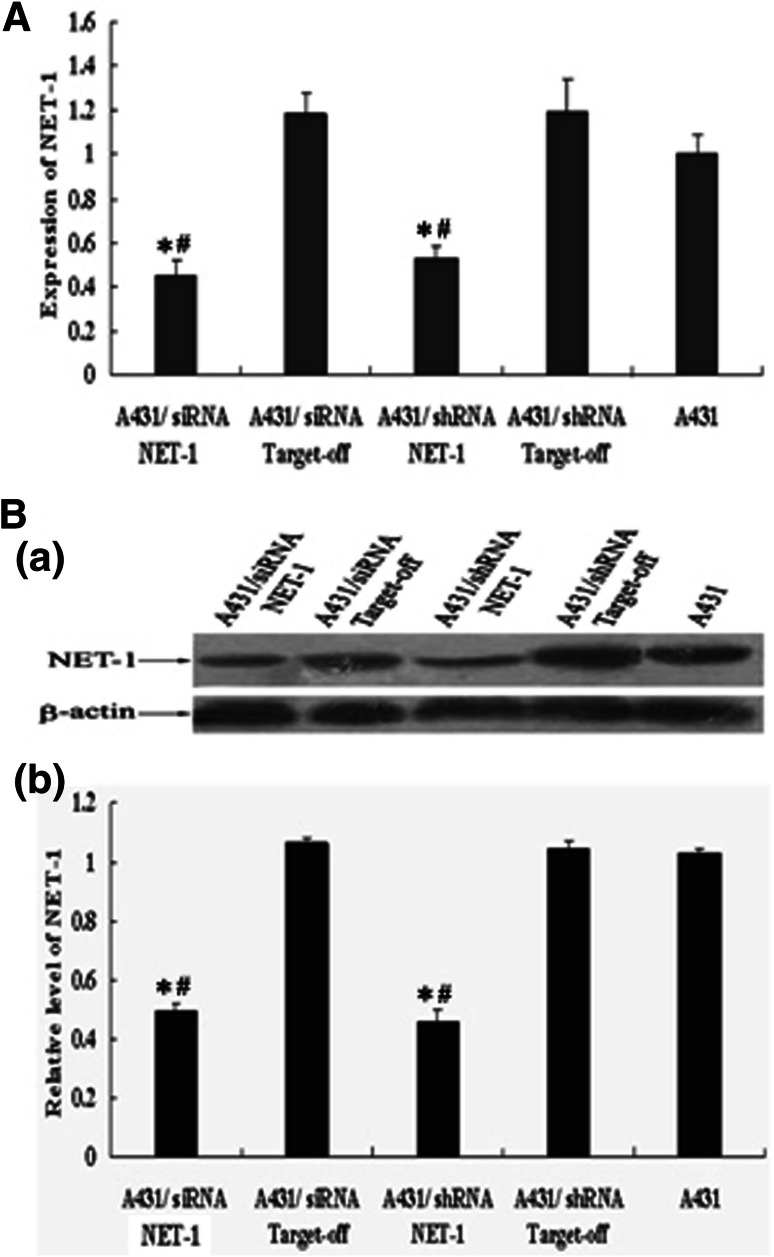
shRNA targeting NET-1 downregulating the expression of NET-1 mRNA and NET-1 protein in A431 cells. **a** RT-PCR analysis for NET-1 expression. GAPDH was used as a loading control. Mean densitometric values ± SEM were calculated and plotted as a histogram. **b** Western blot analysis of the expression of NET-1 protein. *a* The band of NET-1 (38 kD) and β-actin (41 kD); *b* The optical density of NET-1 to β-actin. The untreated group was taken as 1. *Asterisk* statistically different compared to negative control (*P* < 0.05, respectively); *hash symbol* statistically different compared to untreated cells (*P* < 0.05, respectively). Experiments were repeated at least three times

qRT-PCR and Western blotting showed that NET-1 mRNA and protein expression were significantly inhibited in A431 cells transfected with siRNANET-1 or shRNANET-1 (Fig. 3).

### Impact of RNAi on A431 cell proliferation

**Fig. 4:**
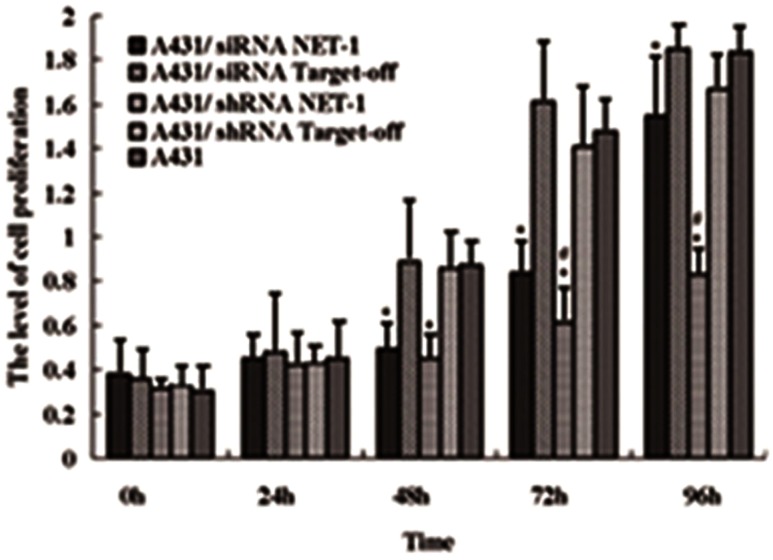
shRNA targeting NET-1 inhibited A431 cells proliferation Cell proliferation was measured at different time-points as described in Materials and methods. The histogram of growth curve showed a statistical difference in A431 cells transfected with shRNANET-1 from 48 to 96 h and transfected with siRNANET-1 between 48 and 72 h. Experiments were repeated at least three times. (*P < 0.01 indicates significant vs. negative control or untreated cells, ^#^ *P* < 0.05 indicates significant vs. A431 cells transfected siRNANET-1)

**Fig. 5:**
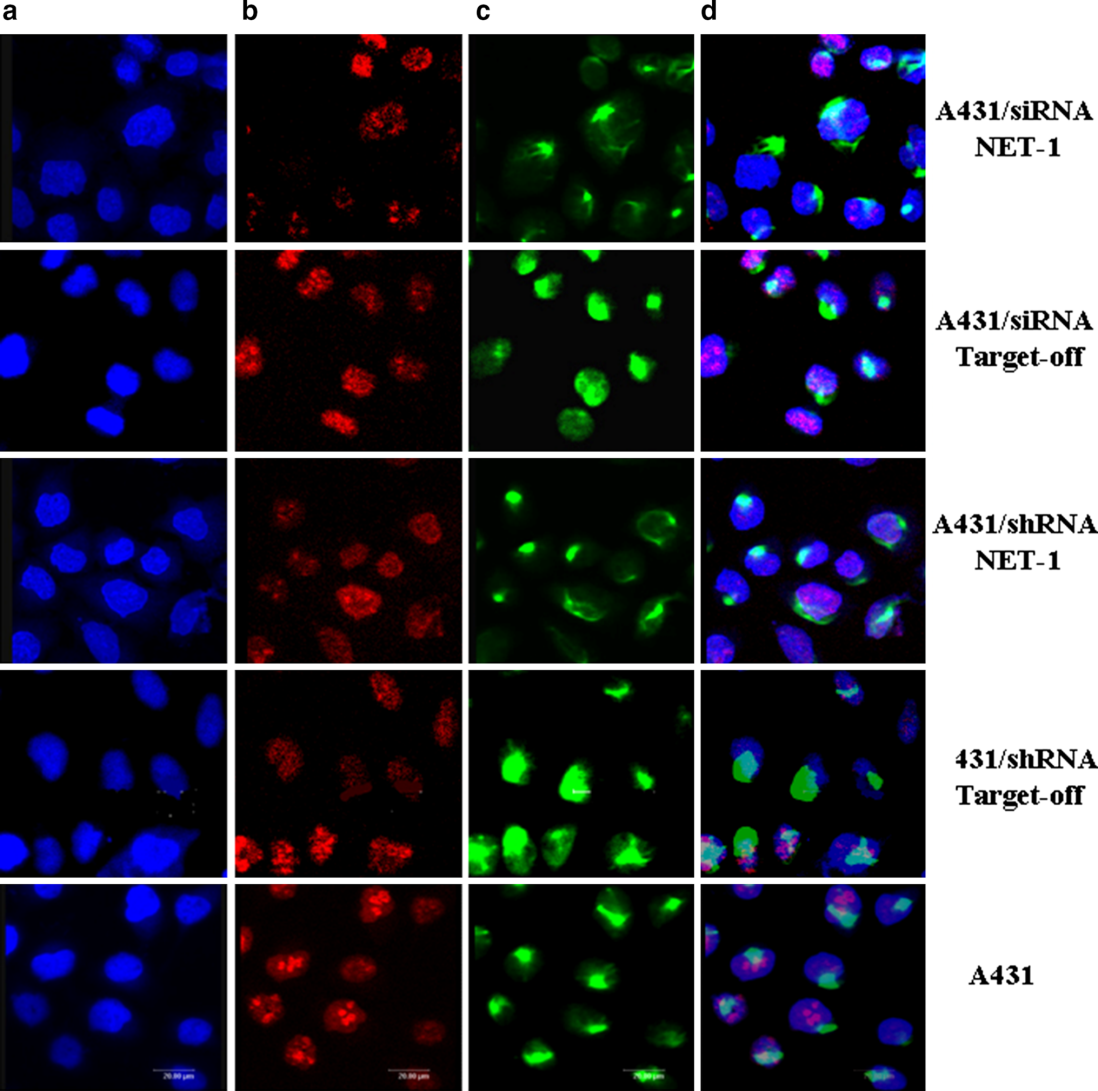
Immunofluorescence staining NET-1 and Ki67 expressed in A431 cells. Nuclei stained by Hoechst in *blue* (**a**), homogeneously Ki67 stained by TRITC in *red* located in the nuclei (**b**), NET-1 stained by FITC in *green* located in the cytoplasm (**c**) in A431 cell lines. Merged figure (**d**)

The transfected A431 cells revealed that both siRNANET-1 and shRNANET-1 were able to significantly attenuate cellular proliferation as compared to negative control or untreated cells (*P* < 0.05). Furthermore the growth curve showed that the inhibiting efficiency in A431 cells transfected with shRNANET-1 was longer than that in siRNANET-1 after 72 h (*P* < 0.05) (Fig. 4). The proliferation index (PI) and cell proportion in every cell cyclic phase were obtained by FCM (Table 4). From Table 4, the cells arresting in G0/G1 or G2/*M* phase were increased, and PI were decreased in cells transfected with siRNANET-1 or shRNANET-1 (*P* < 0.05 vs. the control). In addition, immunofluorescent staining revealed immunoreactivity of NET-1 in cytoplasm,while Ki67 was in nuclei. Two markers were positively correlated in cell (Fig. 5). The expressions of NET-1 and Ki67 were reduced in the cells treated with siRNANET-1 or shRNANET-1 (*P* < 0.05), which indicated that NET-1 gene was involved in cell proliferation.

**Table 4:** Cell cycle percentage and proliferation index (PI) of each group 48 h after transfection

A431 cells transfected with	Cell cycle			
	G0/G1 (%)	G2/*M* (%)	*S* (%)	PI
shRNA NET-1*	70.57	6.78	22.65	29.43
siRNA NET-1*	68.63	10.24	21.13	31.37
shRNA off-T	52.02	14.95	33.03	47.98
siRNA off-T	51.76	15.69	32.55	48.24
Untreated	51.17	11.56	37.27	48.83

* Respectively compared with the control group and untransfected group, the difference was significant in cell cycle and PI (*P* < 0.05)

### Inhibitory effect of RNAi on migration of A431 cells

**Fig. 6:**
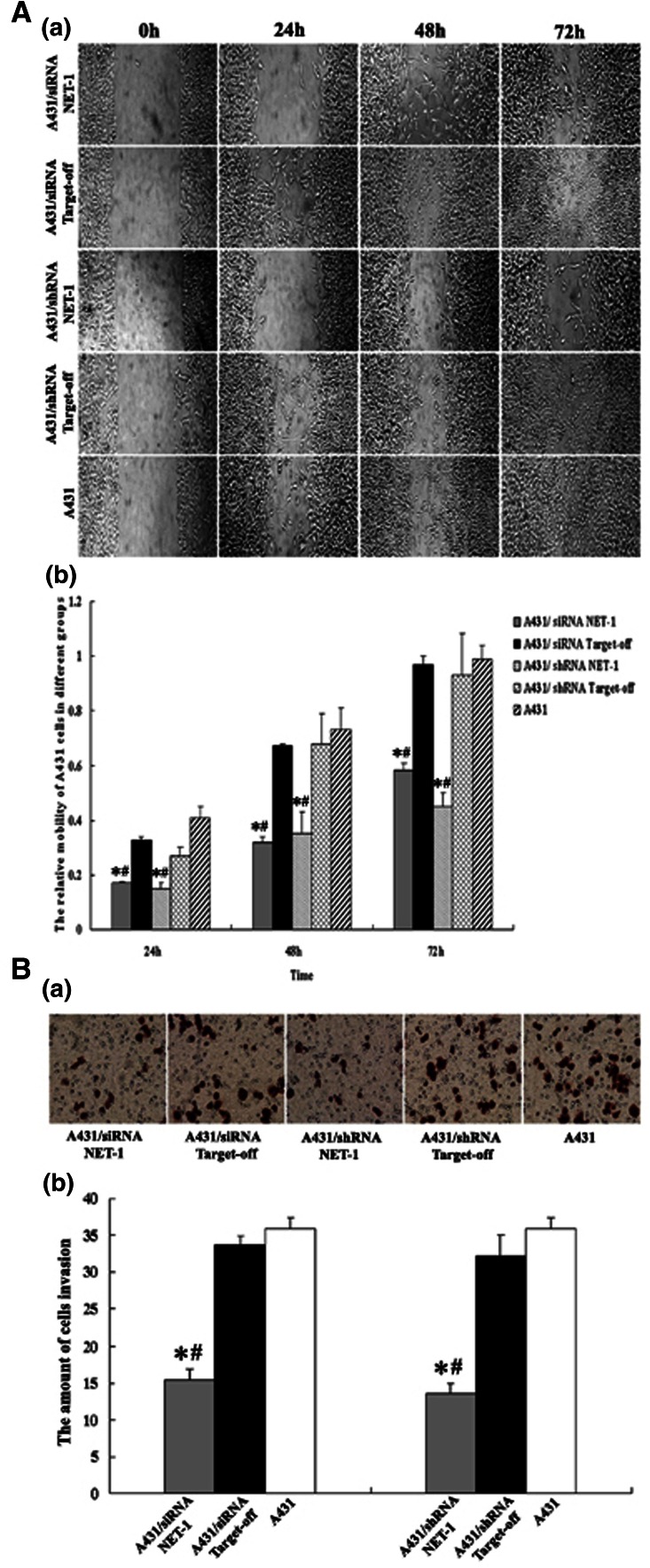
shRNA targeting NET-1 inhibiting the migration of A431 cells. **a** Wound healing assay of the cells migration. *a* Migration cells were measured and photographed under a light microscope at 24, 48 and 72 h as described in “Materials and methods” (×100). *b* Average values of three separate experiments are shown and bars represent the mean ± SE. **b** Transwell small chamber test of cells invasion. *a* Invasion cells in the down room of transwell small chamber were counted under a light microscope at 36 h as described in “Materials and methods” (×200). *b* Quantification of invading cells. The *bars* represent the mean ± SE of three different experiments. *Asterisk* statistically different compared to negative control (*P* < 0.05, respectively); *hash symbol* statistically different compared to untreated cells (*P* < 0.05, respectively)

Wound healing assay and Transwell small chamber test showed that the migration of the cells with siRNANET-1 or shRNANET-1 was significantly affected at 24 h after transfection (*P* < 0.05) (Fig. 6). However, there was no significant difference in NET-1 expression and migration between the negative controls and untreated cells, or between siRNA NET-1 and shRNA NET-1 (*P* > 0.05). But the above growth curve shown the inhibiting efficiency in shRNA was stronger than that in siRNA, so shRNANET-1 was selected to use in vivo.

### Suppression of tumor growth in vivo by NET-1 shRNA

**Fig. 7:**
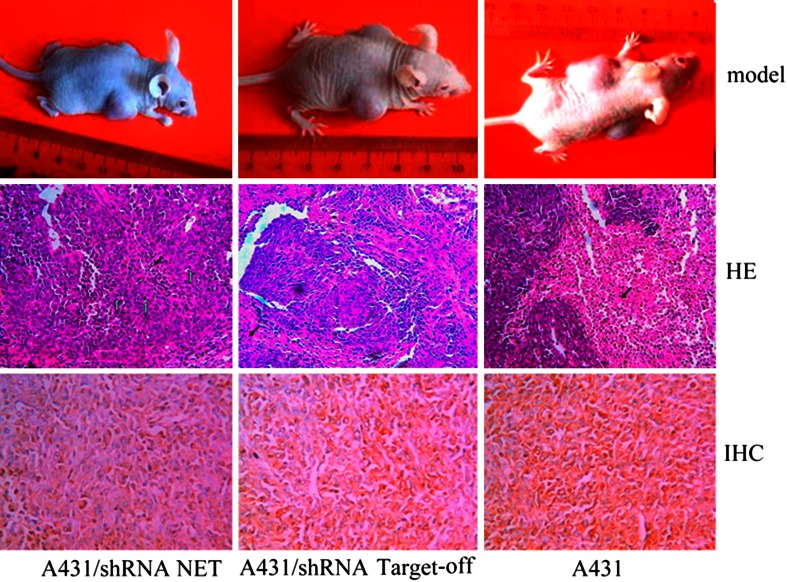
The model and histological manifest of mice xenografts tumor. In vivo, the growth of 20 days after mice xenografts tumor, the tumor was smaller in shRNANET-1 treated tumors than that in untreated cells and the negative control (the first rank). Histologically, cellular apoptosis (*white arrow*) prominently occurred in shRNA NET-1 treated tumors, whereas the large area necrosis (*black arrow*) was prominent in untreated group (the second rank, HE staining, magnification ×100). The expression of NET-1 protein in shRNANET-1 treated tumors was lower than that in untreated cells and the negative control (the third rank, immunohistochemistry stain, magnification ×200)

The volume and weight of tumor xenografts in nude mice were markedly decreased over 50 % of the negative control or untreated cell group at 20 days after inoculation with NET-1 shRNA cells (0.12 ± 0.02 g/192.50 ± 0.40 mm^3^, vs. 0.26 ± 0.42 g/295.19 ± 5.44 mm^3^ or 0.25 ± 0.12 g/276.00 ± 11.04 mm^3^) (*P* < 0.05) (Table 5). Histologically, cancer nests with cellular apoptosis were much obvious in shRNANET-1 tumors; whereas the larger cancer nest with foci necrosis was prominent in untreated group (Fig. 7). As expected, qRT-PCR and immunohistochemical assays revealed that the levels of NET-1 mRNA and protein in shRNA NET-1 xenograft tumors were lower than those in the untreated cells and the negative control (*P* < 0.05), which was consistent with those of human SSCC or in vitro A431 cell study.

**Table 5:** The volume and weight of nude mice xenograft tumors $\left( {\bar{x} \pm S} \right)$ (*n* = 6)

Group	Volume (mm^3^)	Weight (g)	Necrotic area (%)
shRNA NET-1*	192.50 ± 0.40	0.12 ± 0.02	12.5 ± 16
shRNAoff-T	295.19 ± 5.44	0.26 ± 0.42	25.9 ± 31
Untreated	276.00 ± 11.04	0.25 ± 0.12	28.8 ± 09

* Respectively compared with the control group and untransfected group, the difference was significant in tumor volume, weight and necrotic area (*P* < 0.05)

## Discussion

In this study, the expression of NET-1 protein was detected in 60 human SSCC cases, 50 SIN cases and 10 normal human skin samples to identify the correlation between NET-1 and different skin pathological types. In normal epithelial and SIN I–II cells, NET-1 was not or weakly stained predominantly in the basal and spinous layers. In SIN III and SSCC, NET-1 expressed strongly in full epithelial layer with polar disorder. The positive ratio of NET-1 expression was significantly higher in SSCC and SIN III than that in SIN I–II. Spearman grade correlation analysis demonstrated an obvious positive correlation from SIN I–II to SIN III and SSCC. No significant difference between SSCC and SIN III, suggested that SIN III belongs to tumor hyperplasia. And the process of SSCC closely related to upregulated NET-1 expression. Our results strongly supported that the NET-1 overexpression might be an early molecular event in SSCC malignant transformation. In this study, we noticed that NET-1 overexpression implied a cellular response to malignant transformation. Because spindle-cell SSCC is characterized by rapid growth, the poorer differentiation with more mitostic figures, in this study shows that spindle-cell SSCC showed higher expression of NET-1, which suggests that NET-1 may be a marker for cell differentiation phenotype. In addition, NET-1 expression was obviously correlated with tumor infiltrating; it suggests the accumulation of NET-1in cancer cells closely related to the malignant progress of SSCC. These results are similar to Wollscheid V’s investigation [24]. They found that NET-1 gene was expressed in CIN III, cervical squamous cell carcinoma, all undifferentiated cervical carcinoma and adenocarcinoma, indicating that NET-1 gene may be a marker for cervical cancer.

NET-1, the same as TM4SF accumulated in clonal cell surface of SSCC, may transduct signal of cell proliferation, synergistically promotes SSCC invasion and metastasis [18]. Though Co-expression of Ki67 with NET-1 in tumors was performed to evaluate cancer cell proliferation, the results showed that NET-1 might function with Ki67 to increase tumor cell proliferation. Similarly, several reports have correlated the invasive and metastatic potential of cancers with activity and expression of NET-1 in different cancers [5, 7–9, 14, 18].

We used RNAi technique to downregulate NET-1 in A431 cell line. As shown by Western blotting and qRT-PCR, transient transfection with siRNA or shRNA against NET-1 significantly decreased endogenous NET-1 expression and activity, which affected cell cycles, leading to cell arrest in G0/G1 or G2/*M* phase. In addition, the expression of NET-1 in cytoplasm or membrane of A431 cells indicated that NET-1 molecule might receive extracellular signals through transmembrane to function in cytoplasm. The impairment of A431 cell migration capability might be attributed to the downregulation of NET-1. Furthermore, the results in vivo suggested that shRNA NET-1 could significantly downregulate the expression of NET-1 in nude mice xenografts models, leading to stunt the tumor growth and downregulate malignant phenotype of tumor. These results obtained from in vivo were similar to that obtained from in vitro.

In conclusion, our results from human SSCC samples, A431 cell and nude mice model show that the main function of NET-1 gene as “molecular facilitators” may be involved in cell proliferation and migration, carcinoma differentiation and infiltration, tumor development and progress process and may play a tumor-related gene role, or as a candidate gene for diagnosis and a target gene for therapy of skin carcinomas. Therefore, the functions of NET-1 gene and its molecular pathogenesis in influencing biological behavior of SSCC should be furthermore investigated.
